# Heavy Metal Detoxification by Different *Bacillus* Species Isolated from Solar Salterns

**DOI:** 10.1155/2015/319760

**Published:** 2015-10-07

**Authors:** Shameer Syed, Paramageetham Chinthala

**Affiliations:** Department of Microbiology, Sri Venkateswara University, Tirupati 517 502, India

## Abstract

The biosorption mechanism is an alternative for chemical precipitation and ultrafiltration which have been employed to treat heavy metal contamination with a limited success. In the present study, three species of *Bacillus* which were isolated from solar salterns were screened for their detoxification potential of the heavy metals, lead, chromium, and copper, by biosorption. Biosorption potential of each isolate was determined by Atomic Absorption Spectroscopy (AAS), Inductively Coupled Plasma-Optical Emission Spectroscopy (ICP-OES), and Energy Dispersive Spectroscopy (EDS) as the amount of metal present in the medium after the treatment with the isolates. Bacterial isolates, *Bacillus licheniformis* NSPA5, *Bacillus cereus* NSPA8, and *Bacillus subtilis* NSPA13, showed significant level of lead biosorption with maximum of 87–90% by *Bacillus cereus* NSPA8. The biosorption of copper and chromium was relatively low in comparison with lead. With the obtained results, we have concluded that the bacterial isolates are potential agents to treat metal contamination in more efficient and ecofriendly manner.

## 1. Introduction

Heavy metal(s) are widespread pollutants of environmental concern as they are nondegradable and thus persistent [1]. It is well perceived that there is a permissible limit of each metal, above which they are generally hazardous and some are even toxic [2]. It is estimated that over one billion human beings are currently exposed to elevated concentrations of toxic metals and metalloids in the environment and several million people may be suffering from subclinical metal poisoning. In addition, adverse effect of heavy metals includes suppression of the immune system and carcinogenicity, neurotoxicity, mainly in children, and inhibition of the activity of some critical enzymes related to synthesis of vital biomolecules along with accumulation in the body of different organisms causing biomagnifications [3].

Conventional methods like chemical oxidation reduction, adsorption, electrolytic recovery, and so forth are rendered futile due to either financial burden or lack of ecofriendly nature in the remedial process. Despite best human efforts, heavy metals are still increasing in their spread and concentration. This is due to indiscriminate and perilous ways of industrialization in sectors including mining, petrochemicals, and electronics. In 1990s, a new scientific area has developed which could help to recover heavy metals using biological means, that is, biosorption at less expensive manner [4]. The technique of biosorption utilizes the characteristics of living organisms or their biomass to adsorb metals in a commercial manner [5]. This is due to affinity of hydroxylated and carboxylic functional group molecules on bacterial surfaces for heavy metals leading to their adsorption and precipitation. This biosorption is passive nonmetabolic process of binding various chemicals on biomass [6]. Most studies of biosorption for metal removal deal with the use of either laboratory-grown microorganisms or biomass generated by the pharmacology and food processing industries or waste water treatment units [7] and there is only limited amount of information on bioremediation of heavy metal contamination in marine and hyper saline environments using halophilic microorganisms [8, 9].

Therefore, in the present study, we have assessed the biosorption ability of* Bacillus* species,* Bacillus licheniformis* NSPA5,* Bacillus cereus* NSPA8, and* Bacillus subtilis* NSPA13, which were isolated from artificial solar saltpans. The haloalkaliphilic* Bacillus* species present in the solar salterns produce compatible solvents and exopolymers to survive the fluctuating haloalkane conditions [10]. Thus, these extracellular molecules may offer adaptive advantage to the haloalkaliphilic* Bacillus* species to effectively tolerate/remove the heavy metals by biosorption.

## 2. Materials and Methods

### 2.1. Isolation and Biochemical Characterisation of Haloalkaliphilic* Bacillus* sp. from Solar Salterns

For the isolation of the haloalkaliphilic bacteria, 1.0 g of soil sample was collected from solar salterns by employing standard method of soil sampling [11] and was inoculated into 100 mL of the modified nutrient medium with 7% NaCl and the final pH was adjusted to 8.2. After inoculation, flasks were incubated on orbital shaker at 130 rpm with regular monitoring of the turbidity in the media at 37°C. After 48–72 hrs of growth, loop full of culture was spread, plated/pour plated on the nutrient agar (agar 1.5% w/v) plate, and incubated at 37°C for 5 days. Based on the colony characteristics such as form, elevation, and margin, various discrete and distinct colonies were selected and purified. The selected isolates were screened for standard biochemical reactions to establish preliminary identity of the isolates as* Bacillus* species [12].

### 2.2. Molecular Characterization of the Isolates Based on 16S rDNA Gene Sequencing

#### 2.2.1. Genomic DNA Extraction from the Isolates

Genomic DNA extraction was isolated from selected three isolates by following the method described by Sambrook et al. [13]. The isolates were grown in Luria broth for 24 hrs at 37°C. The cells were harvested by centrifugation at 10,000 rpm for 5 min. The pellet was suspended in Saline Tris EDTA (STE) buffer-I (pH 4.0) and centrifuged at 10,000 rpm for 10 min. The pellet was resuspended in STE buffer-II (pH 8.0) and 50 *μ*L of 10% SDS. The cells were left at −80°C for 30 min. To the cell suspension 500 *μ*L of phenol-chloroform was added and spun for 10 min. The supernatant was collected and 100 *μ*L of chloroform: isoamyl alcohol (1 : 1) was added. To the supernatant obtained by centrifuging at 10,000 rpm 1/10th volume of sodium acetate and 2.5 volumes of ice cold 100% ethanol were added and centrifuged for 10 min at 10,000 rpm. The supernatant was removed and pellet was dried for 3 hrs. DNA was resuspended in 20 *μ*L of distilled water.

#### 2.2.2. Amplification and Sequencing of 16S Ribosomal DNA

To identify bacterial isolate of interest, 16S ribosomal DNA was extracted followed by amplification of 16S ribosomal DNA by PCR employing standard protocol [14, 15]. The PCR product was purified and sequenced. Purified DNA product was adjusted to 100 mg/*μ*L concentration in MQ water (pH 8) and sequencing was carried out using forward, internal, and reverse primers in a 313 OXL capillary DNA sequencer utilising thermocycling reaction Big Dye termination version 3.1 in both directions by primer walking method using primers directed to the conserved regions within the gene. The gene sequence obtained was BLAST searched to get homologous sequences followed by phylogenetic analysis of the isolates.

#### 2.2.3. Phylogenetic Analysis

The DNA sequences of the 16S rRNA gene from the isolate of interest were edited manually and BLAST searched individually to find out sequences of homology. The sequences were aligned using the programme CLUSTAL W [16]. The aligned sequences were applied to genetic distance by using neighbour-joining method for phylogenetic inference. Phylogenetic tree was visualized using MEGA tree generation programme.

### 2.3. Heavy Metal Biosorption by the Haloalkaliphilic* Bacillus* sp. Isolates

Heavy metal biosorption is the ability of bacterial cells or components to adsorb, chelate, or precipitate metal ions in the solution into insoluble particles or aggregates which can be removed either by sedimentation or filtration from the solution.

#### 2.3.1. Preparation of Heavy Metal Solutions

Stock solutions of the heavy metals were prepared by using copper sulphate, cadmium chloride, and lead acetate of the respective metals to attain maximum solubility of the metal. The stock solutions were prepared with 1000 ppm concentration of respective metal in milli-Q grade deionised water by compensating for the salt/nonmetallic component (copper sulphate 2.5117 g, cadmium chloride 1.6308 g, and lead acetate 1.8307 g) and stored at 4°C. Standard metal solutions for the metal biosorption analysis were prepared by adding 1.0 mL stock solution to 100 mL of the media giving a final concentration of 1000 ppm.

#### 2.3.2. Assay for Metal Biosorption by* B. licheniformis* NSPA5,* B. cereus* NSPA8, and* B. subtilis* NSPA13

The biosorption of the metals by the isolates was assayed in Erlenmeyer flasks containing 90 mL of metal biosorption medium (NaCl 81.0, MgCl_2_ 7.0, MgSO_4_·7H_2_O 9.6, CaCl_2_ 0.36, KCl 2.0, NaHCO_3_ 0.06, NaBr 0.026, yeast extract 5.0, and glucose 3.0 g/L) [17] added with copper, cadmium, or lead metal solutions having 1000 ppm, final concentration of metal in the medium. To this 10 mL overnight culture of isolates was added having a cell density of 1.5 × 10^6^ CFU/mL. The pH of the metal microbe suspension was adjusted to 6.5 ± 0.02 to facilitate maximum solubility of metal irrespective of the optimal pH for the growth of the isolate. The metal microbe suspension was incubated at 40°C under constant stirring at 150 rpm, for 24 hrs; a control without bacterial culture was also maintained. The biosorption potential was measured as amount of metal removed from the medium by estimating the residual metal concentration using Atomic Absorption Spectroscopy (AAS) and Inductively Coupled Plasma-Optical Emission Spectroscopy (ICP-OES) [18]. All the biosorption experiments were carried out in triplicate and average value was taken from the three readings.

#### 2.3.3. Determination of Residual Metal Concentration Using Atomic Absorption Spectroscopy (AAS)

After incubation, the biosorption of respective metal biosorption by the isolates was measured by removing the cells from the medium by centrifuging at 8000 rpm for 20 min. Standard solutions of individual metals were prepared with varying concentrations in milli-Q water. The standard's absorption of metal solutions was measured by Atomic Absorption Spectroscopy (Shimadzu AA620, Shimadzu, Japan) at wavelengths 324.8 nm, 228.8 nm, and 283.3 nm for copper, cadmium, and lead, respectively. A standard curve was plotted from the absorption of standard metal solutions with concentration against absorption. The supernatant was analysed for residual metal concentration in the bacterial treated and culture free control media. Similarly, the residual metal was also determined by intersecting the absorption of supernatant in the standard curve [19, 20].

#### 2.3.4. Determination of Residual Metal Concentration by Using Inductively Coupled Plasma-Optical Emission Spectroscopy (ICP-OES)

The biosorption capability of bacterial isolates was assayed as above; the biosorption of metal ions was measured by Inductively Coupled Plasma-Optical Emission Spectroscopy (ICP-OES optima 8300, Perkin Elmer, Massachusetts, USA). The ICP-OES was calibrated with standard working metal solutions and blank as above to set limits of detection (10–1000 ppm). The emission lines used for the analysis were 327.393 nm, 228.802 nm, and 340.458 nm for copper, cadmium, and lead, respectively, under Argon plasma with the concentric nebulizer. The residual metal concentration was deduced from internal standard curve produced from standardisation before running the samples and culture free control [21].

#### 2.3.5. Determination of Natural and Loaded Metal Composition Using Scanning Electron Microscopy-Energy Dispersive Spectroscopy (SEM-EDS)

The pelleted* B*.* licheniformis* NSPA5,* B. subtilis* NSPA8, and* B. cereus* NSPA13 cells after biosorption were dried under vacuum and mounted to an appropriate stud surface, thereafter gold-sputtered, and observed and photographed with a Scanning Electron Microscope (Zeiss EVO HD 15, Zeiss, Germany) operating at 20.0 kV. The microscope was equipped with Inca Penta FETx3 energy dispersive X-ray system (England, UK). In order to obtain information on elemental composition of the surface of bacterial cells of metal biosorption studies, the energy dispersive X-ray spectrum of each bacterial isolate against individual metal ion solution treatment was obtained and analysed for elemental composition of the respective metals on cell surface [22, 23].

## 3. Results 

### 3.1. Isolation and Characterisation of Haloalkaliphilic* Bacillus* sp. from Solar Salterns

A total of 14 bacterial isolates were initially isolated from solar saltern soil samples on modified nutrient agar medium. These 14 bacterial isolates were selected on the basis of cultural characteristics such as colony size, colour, form, margin, and elevation and named as NSPA1, NSPA2, NSPA3, NSPA4, NSPA5, NSPA6, NSPA7, NSPA8, NSPA9, NSPA10, NSPA11, NSPA12, NSPA13, and NSPA14. The biochemical characters based on which the isolates were selected for further analysis are presented in Table 1. Based on Bergey's manual of systemic bacteriology, those fitting the description of* Bacillus* sp. and growth characteristics of haloalkaliphilic nature were selected for molecular characterisation [24] and subsequently for biosorption studies.

### 3.2. Molecular Characterisation of the Isolates NSPA5, NSPA8, and NSPA13

**Figure 1:**
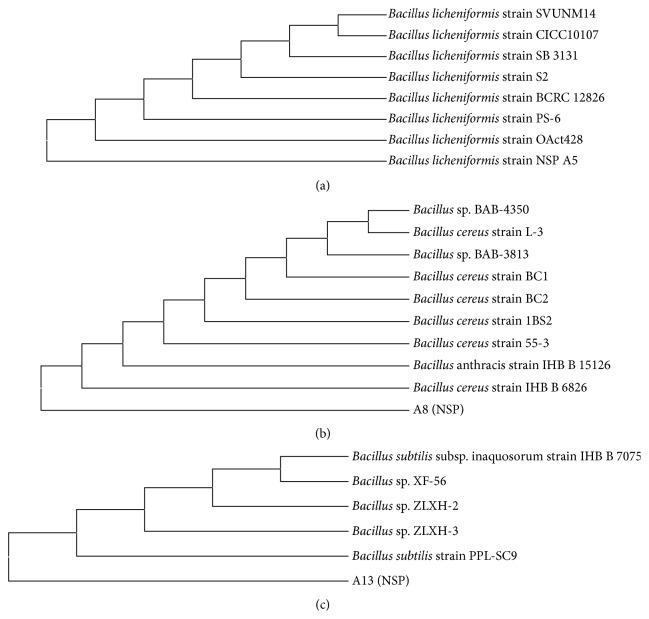
Phylogenetic analysis of the isolates based on 16S rDNA sequence analysis. (a) Phylogenetic tree of isolate NSPA5, (b) phylogenetic tree of isolate NSPA8, and (c) phylogenetic tree of isolate NSPA13.

The selected potential haloalkaliphilic isolates NSPA5 were taxonomically classified using phylogenetic analysis. The amplified 16S rDNA gene using polymerase chain reaction resulted in a single discrete band of a 1.5 kb size in agarose gel. This amplified PCR product was BLAST searched against NCBI Genbank and RDP (Ribosomal Database Project) database 11.0. A distance matrix was constructed based on nucleotide sequence homology using kimura-2 parameter and phylogenetic trees were made using neighbor-joining method (Figure 1). Based on nucleotide homology and phylogenetic analysis, the isolates NSPA5, NSPA8, and NSPA13 showed the highest similarity (99.0%) with* Bacillus licheniformis* (Genbank accession number AB301011) and the nearest homolog was found to be* Bacillus* sp. (Genbank FR823409) and* Bacillus cereus st.* GUFBSS253-84 (Genbank JN315893) 99%, respectively, and the nearest homolog was found to be* Bacillus *sp. BP9_4A (Genbank JN644555) and* Bacillus subtilis st. HS-116* (Genbank JQ062996) 99% and the nearest homolog was found to be* Bacillus subtilis st*. 69 (Genbank JN582031), respectively. The sequences were submitted to Genbank with accession numbers JQ922113, KC686834, and KC686835, respectively, for the sequences of NSPA5, NSPA8, and NSPA13.

### 3.3. Determination of Heavy Metal Biosorption by Using AAS

**Figure 2:**
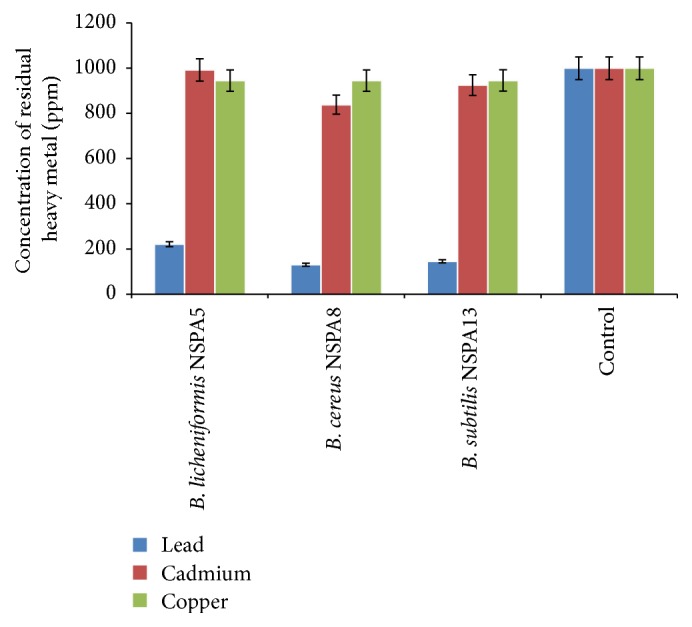
Metal concentration in the medium determined by AAS after removal of the bacteria.

After analysing the treated samples in AAS, the isolate* B. cereus* NSPA8 showed maximum biosorption of the tested metals. The results show all the three isolates were able to adsorb lead at a concentration of 1000 ppm. The metals copper and cadmium were the least adsorbed ones; the metal concentration in the bacterial treated medium is reduced by 78%, 87%, and 86% (221.2276, 130.56505, and 145.2319 ppm) by* B. licheniformis* NSPA5*, B. cereus* NSPA8, and* B. subtilis* NSPA13, respectively, in the case of lead. Copper biosorption was somewhat different as the isolates showed varied biosorption when compared with other two metals; all the three isolates showed very distinct abilities as compared with lead and cadmium. The* B. licheniformis* NSPA5*, B. cereus* NSPA8, and* B. subtilis* NSPA13 reduced the metal concentration of cadmium by 0.8%, 17%, and 8% (992.05, 838.49, and 924.90 ppm), respectively, from the original 1000 ppm concentration. In case of copper biosorption, all the isolates limited themselves to reducing the metal concentration by 6%, 5.5%, and 5.5% (944.97, 945.03, and 945.75 ppm) by the* B. licheniformis* NSPA5*, B. cereus* NSPA8, and* B. subtilis* NSPA13, respectively, showing uniformity in the copper biosorption ability unlike with lead and cadmium. The culture free control showed no reduction in the heavy metal concentration except a negligible decrease in the case of lead (0.01%) (Figure 2).

### 3.4. Determination of Heavy Metal Biosorption by Using ICP-OES

**Figure 3:**
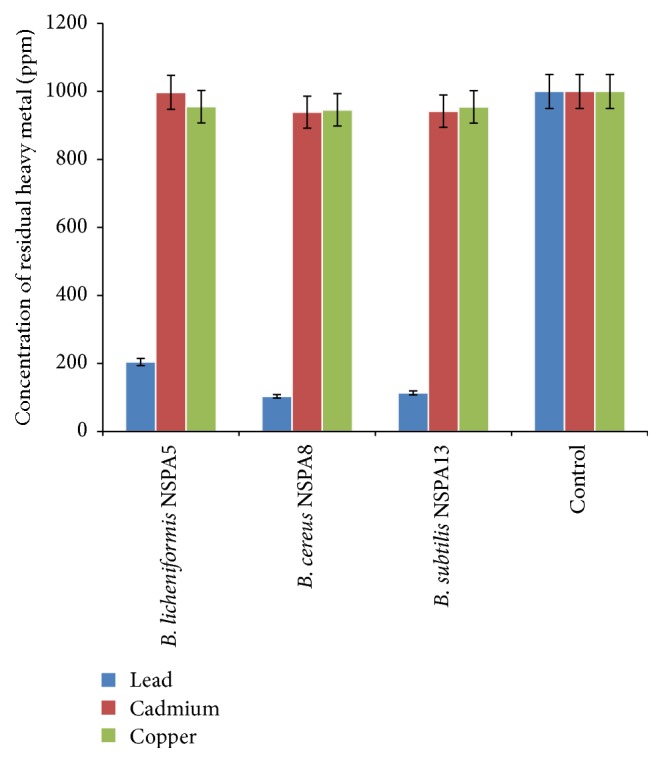
Metal concentration in the medium determined by ICP-OES after removal of the bacteria.

In this method, the emission spectrum is utilized in analysing the metal biosorption ability of the isolates unlike in Atomic Absorption Spectroscopy. Similar to the AAS results, lead was the maximum adsorbed, evident from reduced initial concentration by 89% (1000 ppm to 103.4 ppm) by* B. cereus* NSPA8.* B. licheniformis* NSPA5 and* B. subtilis* NSPA13 reduced initial concentration by 80 and 88% (1000 ppm to 204.4 and 113.85 ppm), respectively. As for cadmium biosorption, the isolate* B. licheniformis* NSPA5 showed negligible adsorption at only 0.3% (997.39 ppm), while the isolates* B. cereus* NSPA8 and* B. subtilis* NSPA13 showed considerable level of biosorption of cadmium reducing the metal concentration by 6 and 5.5% (939.33 and 942.05 ppm), respectively, in contradiction to that of AAS analysis where the same isolates showed diverse biosorption of the cadmium metal ions. Copper biosorption was almost identical to that AAS analysis results with 4.5%, 5.5%, and 4.5% (955.09, 945.84, and 954.55 ppm) of biosorption metal ion from the medium by* B. licheniformis* NSPA5,* B. cereus* NSPA8, and* B. subtilis* NSPA13, respectively. The culture free control showed no decrease in heavy metal concentration except in the case of lead where a negligible decrease was observed (0.01%) (Figure 3).

### 3.5. Scanning Electron Microscopy-Energy Dispersive Spectroscopy (SEM-EDS) to Determine Surface Biosorption of Heavy Metals

**Figure 4:**
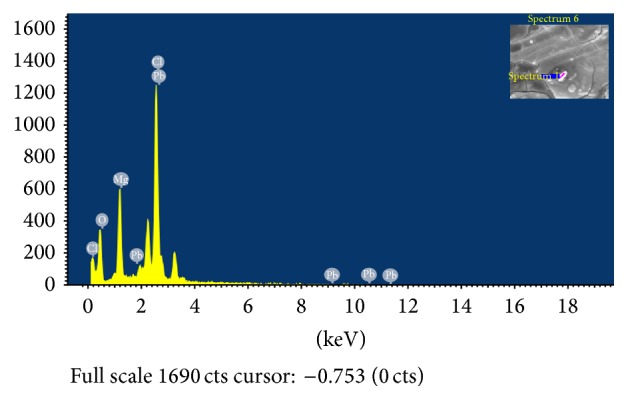
Energy dispersive X-ray spectroscopic (EDS) analysis for elemental composition of lead on cell surface of isolate* B. licheniformis* NSPA5.  ^*∗*^Inset-bacterial cell surface selected for EDS analysis.

**Figure 5:**
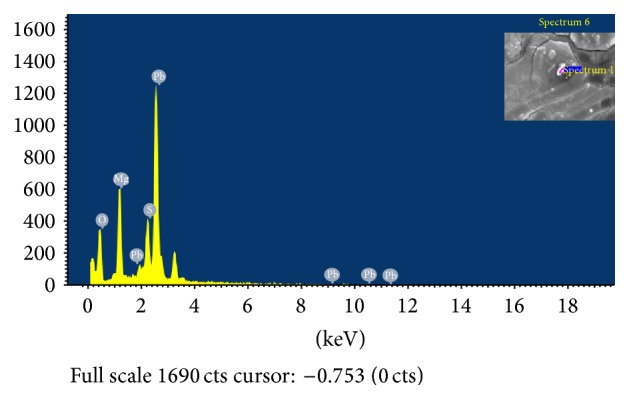
EDS analysis for elemental composition of lead on cell surface of isolate* B. cereus* NSPA8.  ^*∗*^Inset-bacterial cell surface selected for EDS analysis.

**Figure 6:**
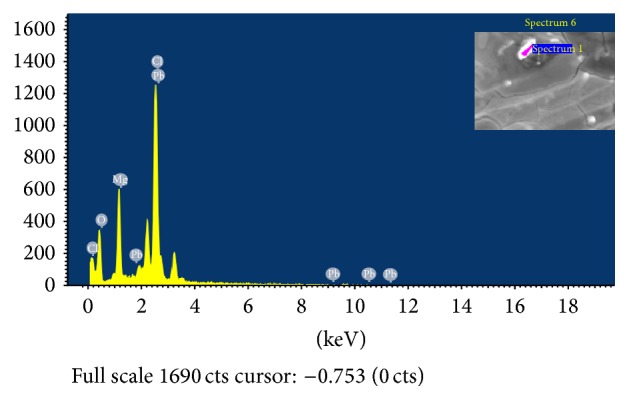
EDS analysis for elemental composition of lead on cell surface of isolate* B. subtilis* NSPA13.  ^*∗*^Inset-bacterial cell surface selected for EDS analysis.

Scanning Electron Microscopy was used to show macro structure of the surface of dry biomass of the bacterial cells. Inca Penta FETx3 energy dispersive X-ray system gave a visible evidence of binding metal ions on the cell wall of bacterial cells. EDS spectral images clearly showed that Cd(II), Cu(II), and Pb(II) ions were adsorbed on the surface of* B. licheniformis* NSPA5,* B. cereus* NSPA8, and* B. subtilis* NSPA13 after biosorption. The EDS spectral images along with SEM images in inset are presented below (Figures 4, 5, and 6).

## 4. Discussion

Increasing industrialization has resulted in an alarming increase in the discharge of heavy metals and other pollutants into the environment including water resources. Microorganisms have been used to remove heavy metals from the environment by various approaches like bioaccumulation and biosorption, oxidation and reduction, and methylation and demethylation [25–28]. The microbe based approach for removal and recovery of toxic metals from industrial effluents can be economical and more efficient in comparison to physicochemical methods for heavy metal removal [29]. Zouboulis et al. [30] reported that certain types of microbial biomass could retain relatively high quantities of metal ions in a process known as biosorption. Various mechanisms have been postulated for the development of metal resistance in microorganisms [31, 32]. However, in general, all these strategies are found either to prevent the entry of metal ions into the cell or to actively pump out the metal ions from the cell [33]. The isolates in the present study showed utmost biosorption of heavy metals tested, particularly lead established by both AAS and ICP-OES analysis; the results are in accordance with the reports of various workers [34–36] and in some instances higher [37]. In our present study, we have achieved up to 87–90% biosorption with moderate extreme conditions when compared with studies involving the same species [38, 39]. The majority of the works compared had prominently acidic pH for the biosorption analysis [40] unlike our present study where slightly acidic medium with pH of 6.5 ± 0.02 was employed without compromising the solubility of the test metals. The reasons for more lead absorption as observed when compared to cadmium and copper was attributed to large ionic size and its heavier atomic weight compared with the rest which enables it for greater interaction with biological components [41, 42]. The metals cadmium and copper were the minimum sorbed metals after lead. The lead biosorption by all the three isolates* B. licheniformis* NSPA5*, B. cereus* NSPA8, and* B. subtilis* NSPA13 stood at 78.9%, 87%, and 85.5%, respectively. The SEM-EDS analysis confirmed the biosorption was different for different metals as reported by Kim et al., [43] onto the cellular surfaces of the bacterial isolates. Chang and Huang [44] showed that lead biosorption modifies groups like carboxyl, hydroxyl, and amino where other metal ions cannot compete offering it more affinity. Copper and cadmium biosorption observed in our study were in agreement with the reports of AL-Garni [45]. However, the biosorption of copper and cadmium was below the optimal reported by studies employing* Bacillus* sp., from different sources [46, 47]. In the present study, very low biosorption of copper and cadmium was observed, when compared with reports involving similar experimental conditions [48]; this phenomenon can be attributed to the fact that cell walls of bacteria contain polysaccharides as basic building blocks which have ion exchange properties and also proteins and lipids and therefore offer a host of functional groups capable of binding to heavy metals. These functional groups such as amino, carboxylic, sulfhydryl, phosphate, and thiol groups differ in their affinity and specificity for metal binding and also in part smaller ionic size, making them less competing in comparison with lead. The SEM-EDS analysis revealed the biosorption mode of copper and cadmium was similar to that of lead; that is, the biosorption was onto the cell wall surface of the bacteria [49, 50].

## 5. Conclusion

Based on the above findings, it was concluded that the isolates,* B. licheniformis* NSPA5*, B. cereus* NSPA8, and* B. subtilis* NSPA13, exhibited maximum biosorption of lead from tested heavy metals. Among the isolated strains,* B. cereus* NSPA8 has showed maximum biosorption of lead (87%), followed by* B. subtilis* NSPA13 (85%) and* B. cereus* NSPA8 (78%) as determined by AAS. Similar results were obtained when determined by ICP-OES. The metal biosorption competence of the isolates was further established with SEM coupled with EDS to ascertain surface adsorption of the metal onto the bacterial cell surface. The present work has proved the ability of haloalkaliphilic* Bacillus* species to treat metal contamination in more efficient ecofriendly manner.

## Figures and Tables

**Table 1:** Biochemical characteristics of the selected isolates.

Biochemical characters	NSPA1	NSPA2	NSPA3	NSPA4	NSPA5	NSPA6	NSPA7	NSPA8	NSPA9	NSPA10	NSPA11	NSPA12	NSPA13	NSPA14
Nitrate reduction	+	+	+	+	+	+	+	+	+	+	+	+	+	+
Citrate utilisation	+	+	+	+	+	+	+	−	+	+	−	+	+	−
H_2_S production	−	−	−	−	−	−	−	−	−	−	−	−	−	−
Indole	−	−	−	−	−	−	−	−	−	−	−	−	−	−
Methyl red test	+	+	−	+	−	+	−	−	+	−	+	+	−	+
Vogesproskauer test	−	−	+	+	+	+	−	+	−	+	−	+	+	+
Oxidase	−	−	−	−	+	−	−	+	−	−	−	−	+	−
Catalase	+	+	+	+	+	+	+	+	+	+	+	+	+	+
Urease	−	+	−	+	+	+	+	−	−	−	−	+	−	−
Starch hydrolysis	+	+	+	+	+	+	+	+	+	+	+	+	+	+
Cellulase hydrolysis	−	+	+	−	+	+	+	+	+	+	+	+	+	+
Lipid hydrolysis	−	−	−	−	−	−	−	−	−	−	−	−	−	−
Casein hydrolysis	−	−	−	−	+	−	−	+	−	−	−	−	+	−
Gelatin liquefaction	−	−	−	−	+^*∗*^	−	−	+	−	−	−	−	+^*∗*^	+
Sucrose	−	−	−	−	+	−	−	−	−	−	−	−	+	−
Fructose	+	+	+	+	+	+	+	+	+	+	+	+	+	+
Glucose	+	+	+	+	+	+	+	+	+	+	+	+	+	+
Galactose	−	−	−	−	−	−	−	−	−	−	−	−	−	−
Lactose	−	−	−	−	−	−	−	−	−	−	−	−	−	−

“−” negative, “+” positive.

“^*∗*^” means delayed positive.
